# The value of a spaceflight clinical decision support system for earth-independent medical operations

**DOI:** 10.1038/s41526-023-00284-1

**Published:** 2023-06-21

**Authors:** Brian K. Russell, Barbara K. Burian, David C. Hilmers, Bettina L. Beard, Kara Martin, David L. Pletcher, Ben Easter, Kris Lehnhardt, Dana Levin

**Affiliations:** 1 Auckland University of Technology, Auckland, New Zealand; 2 NASA Ames Research Center, Moffett Field, Mountain View, CA USA; 3 NASA Johnson Space Center, Houston, TX USA; 4 Baylor College of Medicine, Houston, TX USA; 5 Columbia University, New York, NY USA

**Keywords:** Health care, Mathematics and computing

## Abstract

As NASA prepares for crewed lunar missions over the next several years, plans are also underway to journey farther into deep space. Deep space exploration will require a paradigm shift in astronaut medical support toward progressively earth-independent medical operations (EIMO). The Exploration Medical Capability (ExMC) element of NASA’s Human Research Program (HRP) is investigating the feasibility and value of advanced capabilities to promote and enhance EIMO. Currently, astronauts rely on real-time communication with ground-based medical providers. However, as the distance from Earth increases, so do communication delays and disruptions. Moreover, resupply and evacuation will become increasingly complex, if not impossible, on deep space missions. In contrast to today’s missions in low earth orbit (LEO), where most medical expertise and decision-making are ground-based, an exploration crew will need to autonomously detect, diagnose, treat, and prevent medical events. Due to the sheer amount of pre-mission training required to execute a human spaceflight mission, there is often little time to devote exclusively to medical training. One potential solution is to augment the long duration exploration crew’s knowledge, skills, and abilities with a clinical decision support system (CDSS). An analysis of preliminary data indicates the potential benefits of a CDSS to mission outcomes when augmenting cognitive and procedural performance of an autonomous crew performing medical operations, and we provide an illustrative scenario of how such a CDSS might function.

## Introduction

When living and working in low earth orbit (LEO), astronauts are exposed to radiation, isolation, confinement, altered gravity fields, and a hostile closed environment. Despite a rigorous selection process and exceptional pre-flight health and conditioning status, the rotating crew of the International Space Station (ISS) encounters a myriad of health challenges both on-orbit and post-flight. As a result, space agencies sponsor research for over 30 risks^1,2^ including: radiation carcinogenesis, spaceflight-associated neuro-ocular syndrome (SANS)^3^, muscle changes, renal stone formation^4^, and bone fracture^5^.

**Fig. 1:**
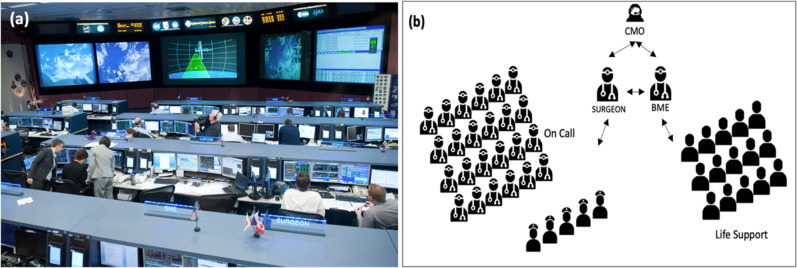
Current paradigm for astronaut healthcare in space. **a** At the NASA International Space Station (ISS) mission control center (MCC) in Houston, TX, the Flight Surgeon (FS) has a dedicated console position labelled SURGEON. Sitting to his left is the biomedical engineer (BME) flight controller. There is often a second medical doctor on console during safety critical operations. Source. JSC2012-E054285 (25 May 2012), Photo credit: NASA. **b** The FS is supported by numerous personnel assigned to provide backup depending on the medical issue. These support personnel include physicians (with an array of subspecialty expertise), nurses, and engineers (responsible for addressing life support capabilities). The FS communicates directly to a crew medical officer (CMO) onboard the ISS.

Health care and medical evaluation on the ISS are managed primarily by a team of clinical practitioners on Earth. Each mission is assigned a dedicated, console-certified, flight surgeon (FS) (Fig. 1a) who maintains direct communication capability with the ISS crew and conducts weekly personal medical conferences (PMCs) with each crew member. The FS monitors and guides the crew through both the mundane and complex medical procedures that arise in real time during the astronauts’ mission. The FS also travels to launch and landing sites to support the crew and help astronauts manage the extreme stressors and medical issues that emerge. A biomedical engineer (BME) flight controller sits to the left of the FS performing real-time monitoring of bioenvironmental data, downlinking and distributing detailed physiological data and scheduling medical activities, among other critical duties.

The FS and BME on the console represent just the visible portion of the ISS ground-based medical support system (Fig. 1b). Since over 100 spaceflight-relevant medical conditions have been identified^6^, a large team of medical experts with a wide range of subject matter expertise supports real-time diagnosis and treatment decisions. Their expertise includes exercise, physiology, psychology, dentistry and a broad range of medical sub-specialties. Environmental control and life support engineers constantly monitor and maintain clean air and water. A crew medical officer (CMO) is always on the ISS. When physician astronauts are onboard, they take the role of CMO. More commonly the role of CMO is assigned to a non-physician astronaut who has approximately 40 h of medical training. On the ISS, the crew is effectively earth-dependent for their healthcare needs.

## Paradigm shift in medical operations

Future missions traveling deeper into space will require an increased reliance on the ability to conduct earth independent medical operations (EIMO). One-way communication delays can be more than 20 min^7^. These communication delays and limited evacuation options will require the crew to detect, diagnose, and treat time-critical medical issues quickly, accurately and autonomously. Autonomous operation is defined here as performing self-directed clinical tasks without realtime mission control center (MCC) support. In an emergency medical situation (acute and life threatening), the crew will need to react swiftly, using locally available information. Preventative and routine health care may be a hybrid system with autonomous data collection and in-vehicle decision support to assist workflow or more critically during periodic lack of communication from planetary alignment, solar wind or equipment failure. Medical inventory will also need to be used strategically in the absence of resupply missions or early return to Earth. Long duration missions beyond LEO will shift the risk balance towards human system failure, i.e. medical risks^8^. Antonsen and Myers^8^ calculated that for a 1195 day mission the crew health index (CHI) would range from 15 to 88% depending on the medical capability provided. Where CHI is defined by Antonsen et al as the percentage of quality-adjusted time lost due to in-flight medical events^8^. On a long duration mission a CDSS could significantly enhance the medical capability and CHI.

A physician astronaut could be included as a crewmember on a deep space mission, but even two or three physicians would not have the breadth of expertise necessary for addressing the full range of potential health-related issues on a multi-year mission. Moreover, if pre-flight crew training is viewed as a fixed resource, additional medical training before a mission means less training time devoted to other critical domains, such as extravehicular activity, spacecraft systems maintenance, or scientific research. Furthermore, there will likely be situations in flight where the CMO is the patient and requires care from clinically novice crew members. A possible solution lies in developing a CDSS to act as an assistive technology to increase the crew’s medical scope of practice (SoP). Here SoP refers to the medical procedures, processes, and actions that an exploration crew can perform.

A CDSS in the vehicle could provide access to broad expertise and knowledge for problem solving, just in time training and refreshment, and guidance to less experienced medical practitioners. The remainder of this paper will describe typical CDSS functions, discuss recent evidence that CDSS can increase SoP and provide a use case that illustrates how a spaceflight CDSS might function.

## A spaceflight clinical decision support system (CDSS)

CDSS refers to technological tools that present healthcare information to users during the clinical workflow^9^. These systems have been used terrestrially for numerous medical care applications from preventive to emergent^10^, in wilderness, military^11,12^, and inpatient/outpatient clinical settings^13–15^. When applied appropriately^16^, CDSS can prevent medical errors and improve health outcomes^9,17^. Examples of terrestrial CDSS functionalities include:

- Medication administration improvements—e.g., reduces prescribing and dosing errors, contraindications through automated warnings and drug-event monitoring^18,19^;
- Rapid interpretation of large volumes of data—e.g., performs complex differential diagnosis, patient probabilistic analysis, provides treatment options and triage based on predicted use of limited resources^20^;
- Streamline documentation processes—e.g., automatically populating clinical notes, constructing auto text shortcuts for lab result interpretation^21^;
- Just-in-time training^22^ and retraining tailored to the user’s skill; and
- Concise logical interactions detailing what to do now, why to do it, how the decision was reached, and what to expect next^23^
- Medical consumable and pharmaceutical inventory tracking

Long-duration exploration missions (LDEM), which are missions into deep space lasting longer than 30 days, present several unique challenges for a CDSS^24^. First, although there are some medical data from short-duration missions to the Moon from the Apollo program (the longest lunar mission was ~12 days), experience with LDEM is lacking^8^. Second, a CDSS must provide real-time support to effectively enable increased crew autonomy for managing anticipated and unanticipated medical conditions. Unanticipated conditions include medical events not previously observed in space or on earth, un-planned, un-resourced or simply not expected at the time of occurrence. Third, the CMO may, or may not, have the expertise required to detect, diagnose, and treat emergent medical conditions. A CDSS should act as a teaming intelligence^25^ to assist the CMO or any user in dynamic, highly complex scenarios, adapting to the acuity of each situation. Given the paucity of clinical evidence for health effects of LDEMs, the system’s role is not to react perfectly but to aid the clinician with a second opinion and provide reassurance that their approach is logical in the face of limited data. Capabilities for a CDSS could include assistive data collection, probabilistic diagnostics, focused real-time guidance, and the provision of diagnostic and therapeutic recommendations. Fourth, spaceflight stressors such as auditory overload, musculoskeletal pain, circadian disruptions^26^, confinement, cultural differences, and elevated carbon dioxide levels all affect cognitive processes^27^ which could affect the crew’s ability to respond to medical events. The CDSS must help the astronauts address a broad range of clinical conditions and must be equipped with a robust data system to augment the users’ knowledge, skills and abilities (KSA) to increase their SoP^28^. A fifth unique challenge for a CDSS is that it should be designed to operate with distance-created communication lags and vehicle-imposed restrictions on computing power, mass, volume, and electrical power^7,29,30^.

A CDSS can increase autonomy by enhancing the user’s ability to independently and appropriately respond to medical events. It should reduce user cognitive workload, help to maintain situational awareness, and support the ability to triage and treat the conditions encountered. The envisioned system should support the diagnostic and treatment workflow, provide management of alerts filtering non-critical events and tailor assessments or recommendations to specific patients, aligned with their medical histories. A CDSS should use communication technologies, data, associated documents and reports, and computational models to assist the CMO. Finally, the system should provide health maintenance guidance to maintain or improve the crew’s quality of life while also preventing illness and injury.

In Table 1, examples of functions of a CDSS for EIMO are categorized by acuity level (emergency, routine, and preventative). For each acuity level a sample scenario is provided, the main goals of care, example CDSS capabilities, and the rationale for CDSS support are provided. Emergency scenarios require clear instructions with priorities decided ahead of time. The airway, breathing, circulation, disability and exposure (ABCDE) approach is one example of an emergency protocol. It is anticipated that a customized version will exist for EIMO taking into account the unique risks associated with deep space and the vehicular environment^31^. Routine clinical treatment is a demanding application for CDSS as cognitive assistance is required for problem solving during diagnosis and treatment. Probabilistic models using Bayesian theory are typically applied to existing clinical workflows to approach the problem in a natural manner. Prevention is challenging for the crew as information is required to inform digital models potentially taking time and attention away from other mission tasks. These preventative models cover various human systems (e.g. renal, cardiopulmonary, musculoskeletal) or exposure risk to determine the likelihood of disease, such as modeling kidney stone risk^4^ or cognitive performance. Ambient detection can be used for prevention to gather information passively thus reducing interaction burden with the crew, for example, measuring movement during task performance^32^ or keyboard timing metrics to assess cognitive performance^33^.

**Table 1:** Clinical scenario grouped by acuity level with example CDSS assistive functionality.

Scenario	CMO goals of care	CDSS assistance	Rationale
Any Medical event	Automation of information flow between vehicle and MCC	Collect, collate and communicate with MCC	Reduce operator time and cognitive load.
Emergency e.g., Cardiac arrest, unexpected life threats Delayed comms to MCC makes this a priority	• Stabilize patient to allow time for full analysis by CDSS, crew, and ground teams and allow further complex treatment • Reduce crew workload • Perform procedures correctly • Acquire and comprehend data from multiple sources	• Easy to access and follow protocols • Prioritized and clutter-free essential information • Electronic procedures; • Just in Time Training • Vital signs^a^ collection and display	• Predetermined protocols for fast-paced, life-threatening scenarios, e.g., ABCDE^32^. • Assist with procedures as required. e.g., intubation • Feedback on success of treatment to inform next step in protocol
Routine – Diagnosis e.g., Headache, Lower back pain Problematic communication delay between each question and answer during a PMC with Earth	• Assess severity • Perform a differential diagnosis • Determine highest probability condition	• Supports clinical workflow • Dynamically adjusts to novice or expert user • Probabilistic engine calculates next best question with highest positive predictive value	• Diagnosis is a complex task • System should support caregiver’s mental model
Routine - Treatment e.g., Headache, Lower back pain Delayed comms make this problematic	• Treatment and care plan • Treat with best practice and minimal use of resources • Manage medications • Manage patient records • Ensure tailored, precision-based medicine • Facilitate efficient and prompt care	• Therapeutic advice based on evidence with ranked options crew can choose from. • Offer a variety of pharmacologic and alternative treatments • Automatic patient record retrieval including allergies, existing treatment and conditions • Provide information on stowage location of medications, supplies, equipment	• A large knowledge base in CDSS is required to give all possible options (e.g. Merck manual^5^) supplied as needed based on diagnosis • Crew personal preferences • Medical errors can easily be mitigated with automatic record retrieval and checking • A simple and extremely valuable task in a space vehicle full of equipment • Trade-off side effects or performance deficits • Options dependent upon limited supply
Routine - Observation Rich data best analyzed on vehicle due to low transmission bandwidth Prompt response to emerging issue	• Assess initial care plan effectiveness • Recognize change in status to higher or lower acuity and adapt plan as needed	• Update previous decisions based on new observations	• Clinical practice treats ‘most likely/severe diagnosis.’ New or persistent symptoms can offer information for new diagnosis or change to emergency scenario
Prevention e.g., Kidney Stones Rich datasets created on vehicle best residing locally due to narrow comms bandwidth	• Anticipate threats to health to reduce medical events over time • Mitigation strategies to enhance and/or prevent decrements in performance • Measure the crew with least possible distractions to operational activities • Accurately record data in medical records	• Prediction of performance decrements or potential medical events • Ambient Monitoring^33^ • Automated record keeping	• Long term issues such as kidney stones, bone loss, behavioral and cognitive deficits can be mitigated with diet, sleep, exercise • Ambient monitoring data collection and analysis reduces workload

*ABCDE* Airway, Breathing, Circulation, Disability and Exposure, *comms* communications, *PMC* Private Medical Conference.

^a^Vital signs include body temperature, pulse rate, respiration rate and blood pressure.

## Quantifying the benefits of a CDSS in EIMO

It is impossible to calculate the exact reduction in risk for every scenario using a CDSS; however, a well-designed CDSS will increase KSA^34,35^. We will describe a proposed method to estimate improved mission risk metrics and medical outcomes across a range of KSA and hence determine the increased operator KSA levels that needs to be achieved with a CDSS. Equivalent terrestrial SoP are used for KSA comparison, shown in Table 2. We show that it is possible to probabilistically stratify the SoP effect on calculated outcomes. Although the mathematical approach is straightforward, the clinical and operational validity of this proposed approach has yet to be peer reviewed. Further detail supporting this preliminary analysis is shown in Appendix A, provided as supplementary information.

**Table 2:** Scope of Practice scale.

SoP Level	Description
0	“No medical training” as a reference for analysis
1	United States National Registry Emergency Medicine Technician - Basic
2	United States National Registry Paramedic
3	Certified Emergency Registered Nurse
4	Medical School Graduate During a Rotating Intern Year (PGY-1)
5	Experienced Attending Physician

NASA’s program for Informing Mission Planning via Analysis of Complex Trade-spaces Medical Database project (IMPACT-MD) uses probabilistic risk assessment (PRA) methodology to estimate mission risk based on medical condition incidence and the on board ability to treat patients^8,36,37^. The analysis assumes that the capability to treat a patient is limited by the maximum level of SoP for cognitive and procedural execution skills. A cognitive skill is categorized as requiring problem solving including decision making. In contrast, a procedural skill requires a physical task following a prescribed procedure, e.g., inserting an intubation tube. Our preliminary analysis showed that increasing SoP reduces mission risk metrics. In addition, cognitive skills had a greater effect on outcomes than procedural skills, as demonstrated in the use case below where lack of early diagnosis could be the difference between an untreated severe condition or a quick resolution. If a CDSS increases SoP it follows that it will also reduce mission risk. For example, with a C-SoP of 1 and P-SoP of 1, roughly equivalent to ISS CMO training, our simulation reference mission of 26 months had up to 3 deaths, but if CDSS can increase C-SoP and P-SoP to level 5, preliminary results show this can be reduced by a minimum of 34%. The preliminary analysis results showed that cognitive SoP above level 3, i.e., medical school graduate, is required to substantially reduce mission risk in human LDEMs. The results are significant and help define an important goal for CDSS for human-machine teaming as it is highly likely a CDSS is required to achieve this level of SoP given the restrictions discussed previously.

In the following section, a use case example involving a sudden medical issue is provided to illustrate how a spaceflight CDSS could be beneficial to an autonomous crew.

## CDSS use case example

While exercising, a crew member (CM) is accidentally struck on the chest due to equipment failure. Other crewmembers activate the CDSS which prompts, “Please state the nature of the medical emergency.” The CM selects “pain” and “left chest.” CDSS identifies several potential diagnoses and precedes to narrow the differential asking the minimum number of questions.

CDSS observes elevated heart rate using ambient sensors, queries medical records and calculates a low predicted risk for heart disease. CDSS requires additional information and alerts the CMO to obtain vitals and an electrocardiogram (ECG) with guidance. CDSS instructs the vehicle communication system to prioritize medical data and alerts the MCC to stand by.

The CMO records “Bruise, Left Chest,” uploads a photograph, and places the ECG leads. CDSS interprets and displays the rhythm as “sinus tachycardia” to the CMO, and transmits results to the MCC. The CDSS reports the most probable diagnosis as chest wall contusion; however, acute coronary syndrome (ACS) remains above the acceptable risk threshold, and the CDSS recommends a blood test for troponin levels. The CDSS checks for allergies and future resource needs, recommending an anti-inflammatory medication, naproxen, and a cold pack for pain management, while indicating storage locations for each. The CMO draws blood and places it in the blood analyzer. The CDSS considers results normal and recommends against a second troponin test to preserve resources.

Thirty minutes later, the CDSS sends a reassessment message to the CM’s tablet asking if symptoms have improved. The CM selects “no,” and CDSS responds: “Please put on a blood pressure cuff and oxygen saturation monitor and tell me what’s wrong.” The CM reports “pain” is the same, but a new symptom, “shortness of breath,” is noted. CDSS notes decreased oxygen saturation (SpO_2_) and increased heart rate.

The CDSS activates emergency mode, sets vehicle illumination to “high,” notifies the CMO and MCC, recommends supplemental oxygen and lung ultrasound to evaluate for a pneumothorax (PTX) or potentially deadly hemothorax. The CDSS guides the CMO through these procedures, notes vital signs have improved and that ultrasound shows no evidence of lung sliding on the left side, suggesting PTX. The CDSS suspends all CM duties and suggests continuous monitoring in the medical bay. CDSS informs the CMO to await recommendations from MCC and recommends just-in-time training (JITT) including “needle decompression” and pigtail catheter insertion.

On Earth, the MCC receives the initial “chest pain” alert from the CDSS and the FS notifies the on-call cardiologist. Both review the ECG and notify the crew they agree with the plan. After the emergency alert, the FS activates the trauma team. Ultrasound images are received and reviewed. They recommend serial ultrasounds and 100% oxygen for 48 h. The CDSS provides JITT and schedules reassessment alerts and monitors vital signs, enabling the CMO to continue normal mission operations between reassessments. MCC monitors patient vitals and the trauma team prepares contingency plans. The FS also meets with mission schedulers to adjust the CM’s duties.

Forty-eight hours later, the CM has improved, and lung sliding has returned. The FS clears the CM for limited duty. CDSS closes the encounter and logs the data in the patient’s chart.

This use case highlights the constant interaction between the CDSS, the CM, MCC, the CMO, and other habitat resources (e.g., the communication system, lighting system, scheduler). Although the clinical approach to this problem may vary across providers, the scenario illustrates the real-time information flow, workload-reduction, and integration of CDSS into onboard and MCC medical operations.

Without a CDSS the CMO needs significantly more expertise and this scenario would evolve very differently. For example, the initial CM request for help pulls both CM and CMO from scheduled tasks. The CMO would reference a database of medical protocols for one that fits the scenario, record a message for mission control, locate the injured CMs medical records, and read through them for any “red flags.” Depending on the chosen protocol, some diagnostic testing may be missed.

When the CMO’s transmission is received, ground teams may or may not have all the information they need (e.g. the ECG and troponin test results). Rather than activating on-call specialists and recommending treatment, they may need to request additional testing and provide limited treatment advice. Depending on perceived severity and available bandwidth, the response may take anywhere from 45 min to several hours to reach the crew.

At this point the patient’s condition is deteriorating. The CMO would need to reassess the CM, search for new protocols, choose which to follow, update ground control, ask other CMs to stop their duties and assist, and monitor for the ground team’s response while the patient struggles to breathe, and suffers increasing pain.

Any new protocol for the worsened symptoms would require a broad range of testing and treatment, decrementing limited on-board resources. When advice from the ground is received, the CMO would need to stop patient care and decide whether to follow the advice, their chosen protocol, or a combination. If combining advice, the CMO would need to decide what to do first. This is a life threatening scenario; if these decisions are incorrect, or slow, the patient could deteriorate into respiratory distress and die.

## Future outlook and summary

LDEMs, compared to LEO missions, have a risk balance shift toward the failure of human systems. Exacerbating this risk balance are the reduced opportunities for resupply, evacuation and access to timely clinical expertise due to communication delays. The crew of LDEMs in deep space must operate more independently than in any previous space mission requiring an increased level of autonomy, which requires early detection, accurate diagnosis and treatment of medical conditions. These new limitations necessitate a shift from the current real-time, ground-based MCC response to medical events to a concomitant increase in the crews’ medical. Given the paucity of data for LDEMs, NASA has analyzed design reference missions to determine medical risk using probability risk analysis. This paper has built upon that work to determine how risk is modulated by cognitive and physical SoP for the crew across a broad range of medical conditions. This paper approached the problem by calculating the improvement in mission outcomes throughout the range of scope of practice both cognitive and physical. The results of the preliminary analysis shown in this paper suggest that increasing the cognitive ability of the crew across a broad range of conditions has the largest improvement on LOCL, need for evacuation, and consumption of resources. Increasing the crew’s medical SoP with an in-vehicle cognitive assistant, such as a CDSS, is a logical step to reduce risk to the crew and enhance the probability of LDEM success.

## Supplementary information

Supplementary Material
