# Supplementary material for: The value of a spaceflight clinical decision support system for earth-independent medical operations
Source: NPJ Microgravity. 2023 Jun 21;9:46. doi: 10.1038/s41526-023-00284-1 (PMC10284846; doi:10.1038/s41526-023-00284-1)
Supplement: Supplementary file 1 — Supplementary Material [file 41526_2023_284_MOESM1_ESM.pdf]

Supplementary Notes: Quantifying the Benefits of a CDSS in LDEM

The analysis described below suggests an estimate of how SoP levels might reduce LDEM medical risk using data from NASA's Informing Mission Planning via Analysis of Complex Trade-spaces Medical Database project (IMPACT-MD) [34,35]. A Clinical Decision Support System (CDSS) which increases the SoP for the crew will directly contribute to the reduction in medical risk by alleviating the need for a medical sub-specialist across many medical conditions.

These results are based on preliminary data from the IMPACT-MD project which is undergoing validation and verification at the time of this publication. A full description of the IMPACT-MD methods will be publicly available through NASA once this process has been completed. This appendix summarizes the methods used to collect the data informing the estimation of risk reduction based on SoP.

IMPACT-MD is a repository of incidence/outcome data, treatment methodology, and medical system resources data for 120 medical conditions considered to be high likelihood and/or of high consequence for LDEMs. It is intended to support the IMPACT probabilistic risk assessment tool for LDEM medical system design and was compiled over 2 years by a team of physicians and other specialties including, epidemiologists, data scientists, biostatisticians, computational modelers, software engineers, and systems engineers. This team reviewed medical literature, terrestrial practice guidelines, spaceflight analogue environment medical data, and medical provider training curricula to develop a list of anticipated LDEM medical conditions, estimate how often each condition might occur and adapt terrestrial standards of care to the LDEM environment.

Condition outcomes affect mission specific metrics including Task Time Lost (TTL) by the affected crew member and does not include the CMO's time, Loss of Crew Life (LOCL), and the probability that a patient will need to return to definitive care (RTDC) i.e., evacuation. Each condition was identified by literature review for both the treated and untreated states. Capabilities were adapted by subject matter experts (SMEs) from terrestrial practice standards and assigned a SoP value by consensus among at least 3 clinicians expert in the specific capability. The SoP was guided by established terrestrial training curricula outlined in **Error! Reference source not found.** in the main text . (e.g. a Primary Assessment is SoP 1 while management decisions for refractory Sepsis are SoP 5). SoP classification values outlined in **Error! Reference source not found.** of the main text are based on well established, curricular requirements for terrestrial providers including national registry emergency medical technician, national registry paramedic, certified emergency registered nurse, first year medical resident, and attending physician. Capabilities were also categorized as procedure execution skills (e.g., performing an ultrasound or inserting a chest tube) or cognitive skills (e.g. interpreting ultrasound images or performing a differential diagnosis). The method made a conservative simplification and assumes a condition can only be treated if all its associated capabilities can be performed by a provider of equal or greater SoP level.

Each outcome metric (e.g. LOCL) was summed across all conditions based upon their treated or untreated values to estimate the outcome's total mission risk. Both the on-board Cognitive SoP (C-SoP) and Procedural SoP (P-SoP) and each condition requires multiple tasks of varying SoP level. Both C-SoP and P-SoP had to equal or exceed the maximum value required by the condition to be considered as 'treated'. If one or both of the on-board SoP scores were less than the conditions' maximum value required SoPs, the untreated values were used. A table was generated for each C-SoP and P-SoP combination for each outcome, and relative risk reduction was calculated using the following equation. Supplementary Equation 1 applies to TTL, RTDC and RTDC.

$$Relative\_mission\_metric(c,p) = 1 - \frac{\text{mission\_metric (c,p)}}{\text{mission\_metric (0,0)}}$$ Supplementary Equation (1)

Where:

c = C-SoP value

p = P-SoP value

mission_metric (c, p) is calculated for C-SoP = c and P-SoP = p ,

mission_metric can be LOCL, RTDC, TTL

The following assumptions and limitations were used to simplify this analysis:

1. The IMPACT-MD was assumed to represent a Mars LDEM even though it was designed for a Lunar LDEM
2. The mission lasts 26 months, with 7 month transits and 12 months on the Martian surface
3. The mission has 3 male and 3 female crew members
4. All conditions can occur to any crew member at any point during the mission except:
   a. Extravehicular Activity (EVA): Each crew member performs 1 EVA per week on the destination surface for a total of 624 EVAs. EVA conditions can only occur while on EVA.
   b. Space Adaptation Syndrome conditions can only occur during the first 5 days after launch from either Earth or the destination.
   c. Gravity Well Adaptation Syndrome conditions only occur within 5 days of landing at the destination.
   d. Surface Operation Conditions only occur during the 12 month stay on the destination surface
   e. Male/Female specific conditions can only occur to 3 of the 6 crew.
5. Mean probability of a condition occurrence is constant for the duration of mission.
6. Capabilities required to treat a condition can only be performed by a provider with equal to or greater SoP level than the maximum SoP capability required. CDSS assistance can be included to increase SoP level.
7. All capabilities must be performed to treat a condition.
8. Treatment resources are unlimited.
9. Task impairments sum linearly and are evenly distributed across the crew.
10. Task impairment only applies to the affected crewmember. It does not include the time lost by the treating CMO.

Supplementary Table 1 Absolute mission metrics using on IMPACT simulated outcomes by on-board Scope of Practice level

	Mean Patient Task Time Lost per Mission by SoP (Hours)					
	Procedural SoP					
Cognitive SoP	0	1	2	3	4	5
0	(4427, 4427)	(4427, 4425)	(4427, 4425)	(4427, 4425)	(4427, 4418)	(4427, 4418)
1	(4426, 3656)	(4426, 3649)	(4427, 3648)	(4427, 3648)	(4427, 3594)	(4427, 3592)
2	(4425, 3637)	(4426, 3631)	(4425, 3630)	(4427, 3629)	(4425, 3574)	(4425, 3571)
3	(4423, 3479)	(4422, 3470)	(4422, 3470)	(4419, 3469)	(4419, 3402)	(4419, 3399)
4	(3964, 1768)	(3957, 1742)	(3957, 1739)	(3950, 1738)	(2914, 1499)	(2915, 1482)
5	(3722, 1505)	(3618, 1475)	(3618, 1472)	(3608, 1470)	(1420, 1202)	(1181, 1182)

	Mean RTDC per Mission by SoP					
	Procedural SoP					
Cognitive SoP	0	1	2	3	4	5
0	(11.52, 11.5)	(11.53, 11.03)	(11.51, 11.03)	(11.52, 11.03)	(11.5, 11.03)	(11.5, 10.59)
1	(11.5, 8.34)	(11.51, 7.76)	(11.52, 7.77)	(11.51, 7.77)	(11.52, 7.74)	(11.5, 7.21)
2	(11.51, 8.33)	(11.51, 7.75)	(11.52, 7.75)	(11.51, 7.75)	(11.52, 7.73)	(11.51, 7.19)
3	(11.53, 6.99)	(11.51, 6.38)	(11.52, 6.4)	(11.51, 6.39)	(11.53, 6.37)	(11.53, 5.81)
4	(14.66, 5.87)	(14.44, 5.26)	(14.44, 5.25)	(14.43, 5.24)	(14.22, 5.23)	(14.22, 4.65)
5	(13.68, 4.02)	(13.32, 3.4)	(13.32, 3.4)	(13.33, 3.39)	(13.06, 3.38)	(2.8, 2.81)

	Mean LOCL per Mission by SoP					
	Procedural SoP					
Cognitive SoP	0	1	2	3	4	5
0	(2.41, 2.41)	(2.41, 2.41)	(2.41, 2.41)	(2.41, 2.41)	(2.41, 2.41)	(2.41, 2.4)
1	(2.41, 0.78)	(2.41, 0.78)	(2.41, 0.78)	(2.41, 0.78)	(2.41, 0.77)	(2.41, 0.77)
2	(2.41, 0.77)	(2.41, 0.77)	(2.41, 0.77)	(2.41, 0.77)	(2.41, 0.77)	(2.41, 0.77)
3	(2.4, 0.38)	(2.4, 0.38)	(2.41, 0.38)	(2.41, 0.38)	(2.4, 0.38)	(2.41, 0.37)
4	(0.15, 0.06)	(0.15, 0.06)	(0.15, 0.06)	(0.15, 0.06)	(0.15, 0.06)	(0.15, 0.05)
5	(0.13, 0.05)	(0.14, 0.05)	(0.14, 0.05)	(0.14, 0.05)	(0.12, 0.05)	(0.04, 0.04)

Supplementary Table 2 Relative mission metrics using on IMPACT simulated outcomes by on-board Scope of Practice level

	Patient Task Time Lost Relative Risk Reduction by SoP					
	Procedural SoP					
Cognitive SoP	0	1	2	3	4	5
0	(0.0, 0.0)	(0.0, 0.05)	(0.01, 0.05)	(0.0, 0.06)	(0.0, 0.21)	(0.0, 0.21)
1	(0.02, 17.42)	(0.01, 17.59)	(0.01, 17.61)	(0.01, 17.6)	(0.01, 18.83)	(0.01, 18.86)
2	(0.04, 17.83)	(0.04, 18.0)	(0.04, 18.02)	(0.04, 18.03)	(0.04, 19.27)	(0.04, 19.34)
3	(0.08, 21.41)	(0.12, 21.61)	(0.11, 21.63)	(0.18, 21.65)	(0.18, 23.17)	(0.18, 23.24)
4	(10.46, 60.05)	(10.62, 60.66)	(10.62, 60.72)	(10.77, 60.76)	(34.17, 66.14)	(34.16, 66.53)
5	(15.92, 66.0)	(18.27, 66.68)	(18.28, 66.76)	(18.51, 66.79)	(67.92, 72.84)	(73.32, 73.31)

	RTDC Probability Relative Risk Reduction by SoP^					
	Procedural SoP					
Cognitive SoP	0	1	2	3	4	5
0	(0.0, 0.0)	(-0.11, 4.07)	(0.06, 4.05)	(0.02, 4.07)	(0.15, 4.1)	(0.13, 7.95)
1	(0.13, 27.48)	(0.06, 32.49)	(0.03, 32.45)	(0.06, 32.46)	(-0.04, 32.71)	(0.12, 37.27)
2	(0.07, 27.57)	(0.04, 32.63)	(-0.01, 32.63)	(0.09, 32.61)	(-0.01, 32.77)	(0.04, 37.5)
3	(-0.07, 39.24)	(0.06, 44.53)	(0.03, 44.38)	(0.05, 44.4)	(-0.1, 44.62)	(-0.13, 49.51)
4	(-27.29, 49.0)	(-25.36, 54.27)	(-25.37, 54.33)	(-25.31, 54.45)	(-23.45, 54.5)	(-23.45, 59.55)
5	(-18.74, 65.03)	(-15.65, 70.41)	(-15.61, 70.41)	(-15.75, 70.49)	(-13.38, 70.6)	(75.66, 75.6)

	LOCL Probability Relative Risk Reduction by SoP[#]					
	Procedural SoP					
Cognitive SoP	0	1	2	3	4	5
0	(0.0, 0.0)	(0.05, -0.11)	(-0.1, 0.06)	(-0.08, 0.16)	(-0.05, 0.13)	(-0.08, 0.38)
1	(-0.14, 67.72)	(-0.19, 67.72)	(0.01, 67.74)	(-0.2, 67.71)	(-0.08, 67.88)	(-0.07, 67.85)
2	(-0.04, 67.91)	(-0.19, 67.86)	(0.01, 67.9)	(-0.19, 67.91)	(-0.11, 67.99)	(0.02, 68.14)
3	(0.15, 84.37)	(0.2, 84.2)	(0.11, 84.23)	(0.09, 84.41)	(0.14, 84.42)	(-0.23, 84.52)
4	(93.85, 97.48)	(93.82, 97.44)	(93.82, 97.42)	(93.83, 97.46)	(93.92, 97.48)	(93.91, 97.73)
5	(94.49, 97.93)	(94.1, 97.84)	(94.05, 97.84)	(94.1, 97.86)	(94.89, 97.95)	(98.19, 98.14)

^*Negative values in this table reflect higher SoPs allowing a reduction in mortality which increases the number of potential evacuation events.*

#*Negative values in this table are an artifact of how the lower bounds are calculated in the simulation.*

Supplementary Table 1 and 2 show the results of a computational simulation using preliminary data from NASA's IMPACT project. IMPACT calculates 3 types of outcomes; Task Time Lost (TTL), Return to Definitive Care (RTDC) i.e., evacuation, and Loss of Crew Life (LOCL). The tables show how these mission outcomes vary by on board cognitive and procedure execution influenced by SoP levels. Supplementary Table 1 shows the absolute risk from the simulation reported in hours of time lost from illness for TTL and the number of occurrences for RTDC and LOCL while Supplementary Table 2 displays the relative risk reduction for these same metrics compared to C-SoP and P-SoP equal to zero. Results are averaged across 200 simulation runs.

There are 2 numbers reported for each scenario which represent a low end and high end estimation for mission risk. The high end risk was calculated using an absolute method. This method assumed that if the on board SoP was lower than the highest SoP required for a given condition no diagnosis or treatment could be given and hence no treatment benefit would occur. The low end method assumed that for a given condition all diagnostic and treatments could be given up to the maximum on board SoP. This means that if the on board SoP is 4 and a condition requires 10 capabilities to diagnose and treat with 9 being SoP 3 and 1 being SoP 5, only the SoP 5 capability could not be performed. In other words 9 out of 10 capabilities *could* be performed and the condition would receive a partial treatment benefit from them. While this is difficult to interpret clinically, it provides a reasonable best case treatment scenario on which to base a low end risk estimate.

For example, for the LOCL section of Supplementary Table 1, the C-SoP 0/P-SoP 0 scenario represents a hypothetical scenario in which no treatment can be rendered for any of the 120 conditions included in the simulated mission. In this case both the absolute treatment method and the partial treatment method demonstrate that 2.41 deaths occurred in the simulation. When the on board SoP is increased to a C-SoP of 1 and P-SoP of 1 representing the terrestrial EMT-B training the low end risk drops to 0.78 deaths while the high end remains at 2.41. SoP-1 is roughly equivalent to the ISS CMO training today. For the optimal situation in which on board C-SoP/P-SoP both equal 5 all conditions are fully treated and both absolute and partial treatment estimations drop to 0.04 deaths. This represents a relative risk reduction for the C-SoP/P-SoP 1 scenario between 0 and 68% and for the C-SoP/P-SoP 5 scenario of more than 98% over the fully untreated scenario. This means that if CDSS can increase an SoP 1 provider to SoP 5 the relative mission risk reduction likely lies between 34% and 98%.

This study does not specify the increase in SoP when a CDSS is used. However it can be seen that the results illustrate increasing SoP reduces the risk of LOCL, RTDC and TTL, i.e., all mission criteria improve in a beneficial direction. The results show SoP levels for cognitive skills have a greater effect on outcomes than procedural skills. The results of this analysis is for illustrative purposes showing the maximum benefit relative to a crew member with no medical training; however, note that it is likely that SoP will be greater than this in real missions. Results show that TTL can be improved by 97% if a CDSS improves both cognitive and procedural SoP to a level of 5. An interesting result from this analysis shows that Procedural SoP had little effect on outcome and Cognitive SoP needs to be greater than 3 to improve mission parameters. Cognitive SoP = 5 will most likely require a CDSS given restrictions on training and the wide medical coverage of likely conditions. Although this method cannot predict actual mission performance, it can provide an estimation of risk and the differences between SoP levels suggests that a high SoP

crew will be better equipped to reduce mission medical risk—regardless of whether this increased SoP is due to human training or automated decision support.
